# Dynamic real-time subtraction of stray-light and background for multiphoton imaging

**DOI:** 10.1364/BOE.403255

**Published:** 2020-12-14

**Authors:** A. Fernández, A. Straw, M. Distel, R. Leitgeb, A. Baltuska, A. J. Verhoef

**Affiliations:** 1 IQSE and Department of Soil and Crop Sciences, Texas A&M University, 4242 TAMU, College Station, TX 77843, USA; 2 Photonics Institute, TU Wien, Gusshausstrasse 27-29/387, 1040 Vienna, Austria; 3 Centro Regional Universitario de Coclé, Universidad de Panamá, Penonomé, Coclé, Panama; 4 Institute of Biology I and Bernstein Center Freiburg, University of Freiburg, Hauptstrasse 1, 79104 Freiburg, Germany; 5 St. Anna Children’s Cancer Research Institute, Zimmermannplatz 10, 1090 Vienna, Austria; 6 Center for Medical Physics and Biomedical Engineering, Medical University of Vienna, Währinger Gürtel 18-20/4L, 1090 Vienna, Austria; 7 alma.fernandez@tamu.edu; 8 aart.verhoef@tamu.edu

## Abstract

We introduce a new approach to reduce uncorrelated background signals from fluorescence imaging data, using real-time subtraction of background light. This approach takes advantage of the short fluorescence lifetime of most popular fluorescent activity reporters, and the low duty-cycle of ultrafast lasers. By synchronizing excitation and recording, laser-induced multiphoton fluorescence can be discriminated from background light levels with each laser pulse. We demonstrate the ability of our method to – in real-time – remove image artifacts that in a conventional imaging setup lead to clipping of the signal. In other words, our method enables imaging under conditions that in a conventional setup would yield corrupted data from which no accurate information can be extracted. This is advantageous in experimental setups requiring additional light sources for applications such as optogenetic stimulation.

## 1. Introduction

Both multiphoton fluorescence imaging [1–4] and optical stimulation, ranging from visual stimulation [5,6] to optogenetics [7–9], are popular tools used in studies of biological processes. A combination of these tools in one experiment yields an attractive setup for precise and minimally invasive *in vivo* (all-optical) manipulation and read-out of cellular activity with sub-cellular resolution [10–13]. One of the main challenges for the combined implementation of all-optical manipulation and read-out methods is crosstalk between the photo-stimulation and the imaging pathways based on the spectral characteristics of the applied and generated signals [13–15]. On the one hand, the spectral content of the photo-stimulation light is chosen to target the absorption spectra of light-sensitive molecules (e.g., opsins, expressed naturally, or through genetic modifications) that can be used to control and modulate cellular activity [13,16–18]. On the other hand, the spectral content of the read-out imaging light is chosen to target the absorption spectrum (or the corresponding multiphoton absorption spectrum) of the fluorescent activity reporter expressed in the targeted cells. In fluorescence imaging a red-shifted spectrum, with respect to the single-photon absorption spectrum, is emitted after excitation. Spectral overlap between the photo-stimulation light, the imaging light, the absorption spectra of the respective light-sensitive molecules and fluorescent reporters, and the emitted fluorescence can cause spurious signals and imaging artifacts. Specifically, spurious signals and artifacts can be due to photo-stimulation of opsins by the imaging light source [13,16], and/or excitation of the fluorescent reporter by the optical stimulation light [5,19].

Thus, the experimental design must take into account the action spectrum targeted for optical stimulation and the absorption and emission spectra of the fluorescent reporters to provide sufficient spectral separation to avoid or minimize crosstalk [9,16,19]. In the field of optogenetics, many efforts have concentrated on reducing spectral overlap through opsin engineering (specifically spectral tuning of their absorption spectra) [12,20–23]. Development of calcium indicators with respect to their absorption spectra also aids to reduce crosstalk [24–26]. Carefully choosing the right combination of fluorescent indicator and opsin helps to minimize spectral overlap, but suppression of crosstalk still remains a challenging task [9,12,19]. An additional strategy applied to minimize opsin activation by the imaging light is keeping the imaging laser power to levels just sufficient for imaging [5,13], but this reduces signal-to-noise ratio. Furthermore, when the cell populations targeted by the photo-stimulation and the imaging beam are spatially well separated, precisely targeting those cell populations to avoid spatial light overlap minimizes the possibility of crosstalk induced by the imaging laser [5]. Fluorescence generated through excitation of the reporter by the optical stimulation light (rather than the light intended for imaging), as well as other spurious signals generated by the stimulation light can be considered as a second type of crosstalk [5,9,10,14,19,27].

In many cases this second type of crosstalk can cause a significant background signal [5,9,19,27]. Artifacts caused by direct detection of the stimulation light have been addressed through temporal separation between stimulation and imaging (e.g. pausing image acquisition during stimulation [28], or stimulation during galvo mirror flyback [5,6]), or spectral filtering [29], or a combination of these approaches [9]. Furthermore, post-processing of the data can be used to correct some artifacts, especially when no clipping of the acquired images occurred. A drawback of reducing the duty cycle of the stimulation light is that it requires higher photon fluxes (stimulation intensity), which can have adverse effects. Pausing imaging during stimulation [28] (or discarding frames compromised by artifacts [13,30]) leads to inherent loss of data. Drawbacks of additional spectral filtering to block narrowband stimulation light overlapping with the fluorescence include the introduction of additional losses to the fluorescence to be detected. Spectral filtering has also been reported to be not sufficiently effective in reducing the stimulation light leaking to the detector [9].

When broadband stimulation light (overlapping with the fluorescence) is used (e.g. from a light emitting diode), or when artifacts are caused by excitation of the fluorescent reporter by the stimulation light, spectral filtering cannot be applied for the suppression of artifacts. Broadband stimulation light is relevant for example for studies of color vision [6]. It may be noted that the spurious signals can also be caused by other stray-light present in the laboratory, like the light originating from computer screens, instrument indicator lamps, or ambient light.

The optical sectioning capability of two-photon optogenetic stimulation [31] allows achieving high spatial precision which has been exploited for precisely targeted stimulation of (groups of) neurons. Used in combination with two-photon calcium imaging, dynamic all-optical manipulation and read-out of neural circuits with cellular resolution *in vivo* was demonstrated [10,13,30]. This allows to implement feedback to dynamically control neural activity patterns, where online analysis of population activity (inferred from two-photon calcium imaging data) is used for neural circuit manipulation ‘on the fly’ [27]. It is worth to note that for this demonstration frames recorded during stimulation are not used for online decision making, and only reconstructed in offline post-processing. Imaging artifacts in the read-out signal due to fluorescent signals generated by the excitation of fluorophores induced by the opsin stimulation laser were observed in experiments using simultaneous two-photon optogenetic stimulation and two-photon imaging read-out of calcium signals [13,27,30]. Corrupted frames were removed or interpolated in post-processing analysis, thereby leading to inherent loss of data [13,27,30]. The removal of measured frames corrupted by imaging artifacts impairs the ability to fully exploit the advantage of simultaneous all-optical stimulation and recording of cellular activity and limits the ability to provide real-time feedback.

We present a new approach to deal with artifacts that affect the recording fidelity of the read-out signals at the detector, such as stray-light or unintended fluorescence. To discriminate against unwanted signals, time-gated fluorescence detection has been used in different microscopy modalities, like time-gated luminescence microscopy [32,33] and some pulsed modalities of continuous wave (cw) stimulated emission depletion (STED) microscopy [34]. The time-gate is introduced to collect the fluorescent signal only during a certain time window after the fluorophore excitation event. Alternatively, a lock-in detection scheme can be applied to suppress uncorrelated background signals [35]. However, an inherent limitation of lock-in detection is that it does not support imaging using only a single laser pulse per pixel.

In this work, we demonstrate an approach that uses time-gated detection combined with a dynamic background subtraction implementation for high-fidelity two-photon fluorescence imaging in the presence of a fluctuating strong optical background. The method takes advantage of the fact that most popular fluorescent markers used for multiphoton calcium imaging have lifetimes in the orders of a few nanoseconds [36], such that the fluorescence signal is being emitted within a few nanoseconds after excitation by an ultrashort laser pulse. Applying excitation at a few MHz repetition rate offers time for the fluorophores to relax to the ground state before the next pulse arrives. Uncorrelated background signals (not correlated to the excitation pulse) that arrive before the excitation laser pulse or several tens of nanoseconds after it can be measured in the absence of fluorescence. Our demonstration is based on a monolithic high-energy Yb:fiber amplifier that can be operated at low-MHz repetition rates, offering plenty of time for most popular fluorescent reporters, with lifetimes in the orders of a few nanoseconds [36], to relax to the ground state before the next pulse arrives. In contrast, ultrafast lasers most commonly used in multiphoton imaging systems operate at 80 MHz repetition rates, corresponding to a time-interval between pulses of just 12.5 ns.

To test the effectiveness of this method to suppress uncorrelated background signals during simultaneous optical stimulation and optical read-out, we apply a strong modulated background (orders of magnitude stronger than the weakest fluorescence features in our sample) that spectrally overlaps with the fluorescence emission spectrum. This configuration is chosen to emulate experiments where two-photon fluorescence microscopy is combined with photo-stimulation techniques like visual stimulation and optogenetic techniques. We implement real-time background subtraction using readily available field programmable gated arrays (FPGA) without the need for lock-in detection schemes. This approach is compatible with single pulse per pixel imaging. We compare this new approach to ‘conventional’ multiphoton imaging where the signal from several laser pulses is integrated electronically before sampling and show that our approach can eliminate background artifacts even in cases when this is not achievable with ‘conventional’ imaging and post-processing. Our new approach can be used to improve feedback control for ‘on the fly’ optical stimulation, since it offers the possibility to utilize frames otherwise corrupted by photo-stimulation artifacts. Moreover, our approach offers new possibilities for experiments where uncorrelated background light prevents accurate recovery of the signal of interest, and it removes the requirement to temporally restrict photo-stimulation during readout in other experiments especially where spectrally broad stimuli are used.

## 2. Methods

### 2.1 Optical setup

**Fig. 1.**
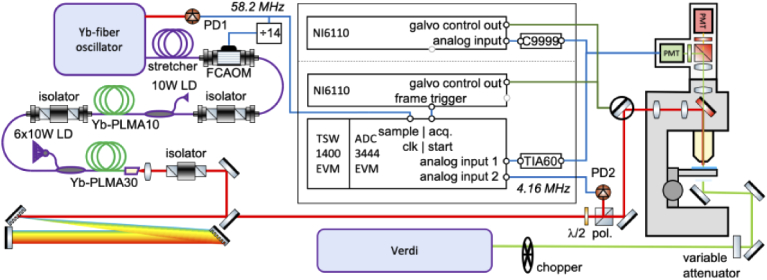
Schematic of the setup. The Yb:fiber laser (left) is seeded with an Yb:fiber oscillator running at a repetition rate of 58.2 MHz. The pulse train from an auxiliary output of the oscillator is detected with a photodiode (PD1), which provides a trigger for the delay generator card used to provide the driving signal to the fiber coupled acousto-optic modulator (FCAOM), and the data-acquisition electronics. After the FCAOM, the light is amplified. A grating compressor (two 1480 lines/mm gratings at 46.5° incidence angle, 50 cm separation) compresses the pulses from the Yb:fiber laser and precompensates the dispersion of the components in the excitation light path to and in the microscope. The microscope (right) consists of a modified Zeiss Axioplan equipped with photomultiplier tubes (PMT) for fluorescence detection and a pair of galvanometric mirrors for laser scanning. See the methods section for more details.

**Fig. 2.**
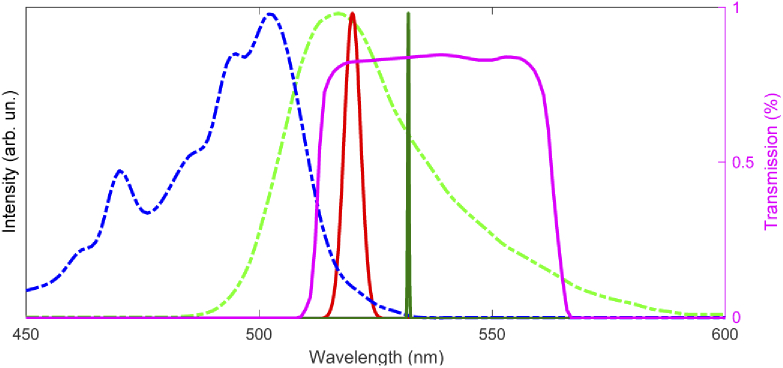
Absorption (dash-dotted blue line) and emission (dash-dotted green line) spectrum of Dragon Green fluorescent beads. The solid red line shows the second harmonic spectrum of the Yb:fiber laser used for two-photon excitation of the beads. The solid dark green line shows the spectrum of the green laser illuminating the sample from below. The solid magenta line shows the transmission of the interference filter in front of the PMT detecting the fluorescence.

Our custom-built microscope for two-photon fluorescence imaging is sketched in Fig. 1. It uses a custom-built ultrashort Yb:fiber laser suitable for two-photon calcium imaging using green and red fluorescent calcium indicators. The Yb:fiber laser delivers 180 fs pulses at 1040 nm and 4 MHz pulse repetition rate. The laser uses an acousto-optic modulator (AOM) to reduce the repetition rate of the amplifier compared to the 58.2 MHz repetition rate of the seed oscillator. A more detailed description of similar laser architectures can be found in [37,38]. The 4 MHz repetition rate corresponds to a time separation of 250 ns between pulses. A continuous wave laser with a 532 nm wavelength (Coherent Verdi V5), modulated with a mechanical chopper, is used for creating a strong artificial fluctuating background. For our proof-of-principle experiments, two photon fluorescent images were obtained from fluorescent beads (Dragon Green fluorescent polymer, mean Ø 15.45 µm, Bangs Laboratories Inc) sandwiched between a microscope slide and a cover slip. The excited state lifetime of the fluorescent molecules embedded in the slide is ∼1.3 ns, which is much shorter than the time separation between pulses. Note that the excitation and emission spectrum of the Dragon Green beads (see Fig. 2) are quite similar to the excitation and emission spectrum of the genetically encoded calcium indicator GCaMP, while the fluorescence lifetime of GCaMP is about 2.8 ns [39], which is twice as long as for the Dragon Green beads, but still two orders of magnitude shorter than the time in between two pulses in our system.

We use a Zeiss Axioplan microscope frame modified for two-photon laser scanning microscopy. The upper illumination part is removed, and the epi-illumination dichroic beamsplitter is replaced by a 45° ultrafast laser mirror with high reflectivity in the range from 1010 nm to 1080 nm, which also has a high transmission for visible light. A Leica PlanFluotar 40X 0.7 NA infinity corrected dry objective is used to focus the pulses on the sample. A custom-made adapter is used to mount a pair of photomultiplier tubes (PMT) on the camera port on the top of the frame. The fluorescence light from the sample is collimated by the objective, transmitted by the ultrafast laser mirror and imaged onto the original camera plane of the microscope by the Zeiss tube lens. A lens with f=50 mm (Thorlabs LA1131-A) is placed about 5 cm above the original camera plane, such that the fluorescence light is collimated again, i.e., the original Zeiss tube lens and Thorlabs lens form a Keplerian telescope with a magnification of 0.3. The collimated fluorescence is divided into a red and green channel with a 45° long-pass dichroic mirror (high transmission > 590 nm, high reflection < 590 nm). The photocathodes of the PMTs are located about 5 cm away from the center of the lens, such that the fluorescence from different locations in the sample image on the same area of the PMT. Directly in front of the PMTs, an additional interference filter is mounted (‘red’ filter 645/75, ‘green’ filter 535/50 – central wavelength/width, nm). In the current work, only the ‘green’ detection channel is relevant.

The upper illumination parts of the microscope (broadband light source, shutter, lenses, filters and iris) are replaced by the laser-scanning setup, consisting of two galvanometric mirrors (Thorlabs GVSM002/M) and a telescope. The telescope consists of a f=75 mm lens (Thorlabs LBF254-075-B) and a f=300 mm lens (Thorlabs LA1541-B), with the focus of the first lens located in-between the two galvanometric mirrors. The focus of the second lens (taking into account the glass components routing the upper illumination path to the dichroic) is located at the back aperture of the objective.

In order to maximize the scanning speed of the beam over the sample with our system, the galvanometric ‘x’-mirror is driven with a sinusoidal waveform with a 2 ms period (i.e., a single line is scanned in 1 ms), instead of a sawtooth waveform. Using a sawtooth waveform for the x-scanning limits the speed at which the mirrors can be operated. The ‘y’-mirror is driven by a slower linear waveform. The high-frequency sinusoidal waveform allows for a maximum scan amplitude of ∼1.5°, allowing for a field-of-view (FOV) of ∼300×300 µm^2^.

The beam size from the Yb:fiber laser (operated with ∼500 mW output power at 4 MHz repetition rate) is adjusted with a small telescope such that the back aperture of the objective is slightly underfilled, slightly enlarging the focus in order to avoid saturating the two-photon induced fluorescence. A half-wave plate and polarizer in the beam path are used to adjust the laser power on the sample to ∼20 mW. A small portion of the remaining laser power is directed to a photodiode that is used for signal monitoring, and the rest is dissipated on a beam dump.

For the proof-of-principle demonstration of our background correction method, we illuminate the sample from below with the aforementioned 532 nm beam. The 532 nm wavelength was chosen to be within the spectral window of the two-photon induced fluorescent signal of the imaged beads (as depicted in Fig. 2), and also to fall well within the transmission window of the filter in front of the PMT of the ‘green’ detection channel. Note that in all-optical stimulation methods where two-photon excited fluorescence microscopy is used in combination with optogenetic activation, fluorescent calcium indicators and opsins are carefully chosen in such a way that stimulation and recording (fluorescence emission) spectrum peaks do not coincide (rather the spectral separation between those spectra is chosen to minimize crosstalk), i.e. our choice of wavelengths represents a worst-case scenario. In order to demonstrate the effectiveness of our method in a single image, we modulate the light from the 532 nm green laser with a mechanical chopper at a frequency of ∼20 Hz. Illumination of the sample with the green laser was achieved by removing the condenser below the sample and replacing it by an inclined diffuse scattering surface, and the area illuminated by the green laser is located a few mm below the microscope slide. It would also be possible to use a mirror instead of the diffuse scattering surface, but this would cause the green light to be focused on the photocathode of the PMT, rather than being spread over the entire active area, which can lead to damage of the photocathode. A variable neutral density filter in the 532 nm beam path is used to allow control of the amount of background light detected by the PMT. The power of the modulated 532 nm beam was adjusted such, that during the times it was passing through the chopper the amplified signal from the PMT exceeds the maximum pixel value on the image in the ‘conventional’ imaging mode (as described in the following section), i.e., the signal is clipped and observed as white regions in the resulting image. As a result, accurate reconstruction of the image using post-processing is not possible. During the times that the 532 nm beam is blocked no background is observed in the recorded image (black stripe regions in the recorded images). The same illumination level conditions were then used while imaging using the mode where real time background suppression was implemented (see the description in the following section).

The low pulse repetition rate (4 MHz) of our home-made laser allows for increasing the energy delivered to the sample by a single laser pulse, while keeping the average power below the damage threshold of the sample. Reducing pulse repetition rate while maintaining average power means that the average fluorescence signal per pulse is higher than what would be obtained with the same average power (and central wavelength and pulse duration) with a pulse repetition rate around 80 MHz (most ultrafast lasers used in commercial multi-photon microscopes operate with a pulse repetition rate of 80 MHz). The fluorescence signal induced by a single laser pulse is increased by about 400 times. The second consequence of using a pulse repetition rate significantly lower than the most common 80 MHz repetition rate is that the fluorescence signal induced by a given laser pulse has decreased back to the base level long before the next laser pulse interacts with the sample. A consequence of the higher fluorescence signal per laser pulse is also that the gain setting (bias voltage) for the PMT can be significantly lower in order to optimally cover the dynamic range of the data-acquisition system. This also reduces the sensitivity of the detection system to uncorrelated stray-light. At high gain settings, it is commonly observed that stray-light (for example originating from room lights) causes the current-overload protection of PMTs to switch off the PMT power. The photocurrent caused by stray-light is significantly lower when the PMT gain setting is significantly lower, thus allowing for a much higher amount of stray-light reaching the PMT before the current-overload protection activates.

### 2.2 Data acquisition electronics

For the work presented here, we compare the ‘conventional’ acquisition mode (which we refer to as acquisition mode 1) that is most widely used in two photon fluorescent microscopy with the method proposed in this work (which we will refer to as acquisition mode 2). The two different data-acquisition modes were compared using the same low laser repetition rate and average power. Our laser also allows operation at 58.2 MHz, which is much closer to the 80 MHz repetition rate of lasers typically used in multiphoton imaging systems. Tests at 60 or 80 MHz repetition rate and the same average power would however give a substantially lower signal-to-noise ratio than with 4 MHz in acquisition mode 1 and the higher repetition rate is not suitable for acquisition mode 2, therefore this configuration was not tested.

Acquisition mode 1 uses electronics frequently found in existing multiphoton imaging setups: The fluorescent signal used for imaging was detected with the PMT and amplified using a transimpedance amplifier (Hamamatsu C9999, 10 MHz bandwidth), and sampled at 1 MS/s (i.e., 1 million samples per second) with an analog-to-digital converter (ADC, National Instruments PCI-6110). A low-pass filter in between the transimpedance amplifier and the ADC ensures that the detection bandwidth matches the sampling rate.

Acquisition mode 2 uses electronics with faster instrument responses, i.e., the transimpedance amplifier is replaced by a 60 MHz bandwidth device (Thorlabs TIA-60), and the ADC is a 125 MS/s, 4-channel digitizer (Texas Instruments ADC3444EVM) interfaced to the measurement PC with an FPGA communication board (Texas Instruments TSW1400EVM). For convenience we used the 58 MHz repetition rate of the seed oscillator of our Yb:fiber laser as sample clock, resulting in a sampling rate of 58 MS/s for each channel. While this is lower than the maximum sampling rate possible with the used ADC card, it is important to derive the sampling clock from the laser and the oscillator pulse train provides the most convenient trigger. Two channels are used to record the light detected by the PMTs, one for the ‘green’ channel, and one for the ‘red’ channel (although not in use in the current experiment), and a third channel to record the pulse train from the Yb:fiber amplifier (measured using PD2, Fig. 1). This last signal is used to verify that no data samples are missed, as well as to provide a reference signal to determine the timing of the fluorescence signal. Alternatively, a signal taken from the laser control electronics synchronizing the pulse-picker in the Yb:fiber laser can be used for this task. The laser control electronics divides the pulse repetition rate of the oscillator by 14, to yield a ∼4 MHz control signal for the pulse picker with extremely low timing jitter. Thus, the measured signals from the ADC can be divided up in 14 sets of samples, each set of samples timed at a fixed delay from the laser pulses interacting with the sample.

In both modes the galvanometric mirror scanning is controlled by the PCI-6110 board. In the second mode, the PCI-6110 provides an acquisition start-trigger to the ADC3444EVM board for each frame to be recorded and line triggers that are used to establish line separators in the data stream. The PMT bias voltage (gain) is adjusted such that the strongest fluorescence signal (without background light) amounts to about 30% of the pixel saturation value (the ADC measurement range maximum) when imaging with acquisition mode 1, and the same PMT bias voltage is used when imaging with acquisition mode 2. Having the maximum fluorescence signal adjusted to about 30% of the ADC range maximum allows to accommodate increases in fluorescence intensity of calcium indicators as a result of neuronal activity without causing signal clipping. Because of the inherently different amplification of the transimpedance amplifiers used in the two acquisition modes, in acquisition mode 2 the same fluorescence signal converts to roughly 20% of the pixel saturation value.

In our setup an older workstation controls the imaging, therefore the sampling rate of the NI-6110 data-acquisition card was limited to 1 MS/s and the number of lines per frame was limited to 400 lines. While the FPGA and ADC of the Texas Instruments evaluation boards are specified to allow a high throughput of the acquired data, the data-transfer to the PC is limited by the USB communication chip, thus each frame had to be stored on the local memory of the card and transferred after completion of the frame, which took considerably longer than the time needed to acquire the frame. It is important to note that most current multiphoton imaging setups are equipped with high-throughput data-acquisition systems capable of real-time data-transfer and high-end workstations that can handle data streams of several input channels sampled at 125 MS/s or even higher rates. This means that in order to implement this method in state-of-the art microscope systems the detection electronics does not need to be replaced provided the two-photon fluorescence excitation sources can be adapted to allow for an appropriate repetition rate to allow for the fluorophore to decay back to the ground state between laser pulses, for example by using a fast AOM in the beam path.

### 2.3 Data processing

**Fig. 3.**
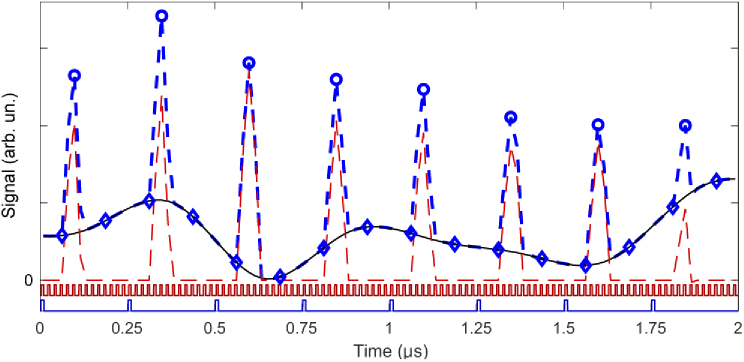
Timing choice of the measured fluorescence and background signals. Dashed blue line – signal trace on the ‘green’ PMT over a time span of 8 laser pulses, in the presence of background light. Blue circles – fluorescence signal samples. Blue diamonds – background samples. Thin black line – spline through the background samples. Dashed red line – signal with background subtracted. Dark red line – sample clock triggers. Dark blue line – pulse clock triggers.

In the second data-acquisition mode, which we used to implement dynamic background suppression, typically only one or two of the 14 samples measured per laser pulse contain two-photon-excited fluorescence signal. The other samples will contain background/stray-light signals if present. In the absence of background/stray-light, the image can be formed by just taking the values of the samples that contain signal. When stray-light is present, the image quality will be degraded by the stray-light reaching the detector, and this can be improved by subtracting the value measured in one of the samples containing only stray-light (or the average of several samples containing only stray-light). In our demonstration we used two samples, one ∼30 ns before the peak of the fluorescence, and one ∼80 ns after. Figure 3 shows an example for the choice of the timing of the measured fluorescence and background signals, showcased for a signal seen on a PMT over the time span of 8 laser pulses in the presence of background light. In contrast, in the first (conventional) data-acquisition mode, the signal is integrated over 4 laser pulses – only one sample is acquired every microsecond – which does not allow measuring the uncorrelated background.

Because we use a sinusoidal driving signal for the ‘x’-galvanometric mirror, the raw images are distorted along the horizontal direction. Moreover, because the 500 Hz driving frequency is more than the maximum frequency supported by the mirror, the actual motion of the mirror is slightly anharmonic. Therefore, we resample the raw measured images (1000×400 pixels for the first data-acquisition mode, 4159×400 pixels for the second mode) to obtain an equidistant 400×400 pixel grid. This is done by calculating the actual beam position for every measured sample, using the ‘set’-position following the programmed sinusoidal signal and a second sinusoidal (sin^2^) term representing the mirror inertia (having a phase lag of ∼π/30, and an amplitude of ∼16 pixels). The anharmonicity of the motion can be avoided by using a slower scan speed or by the use of a resonant mirror, for example.

## 3. Results

**Fig. 4.**
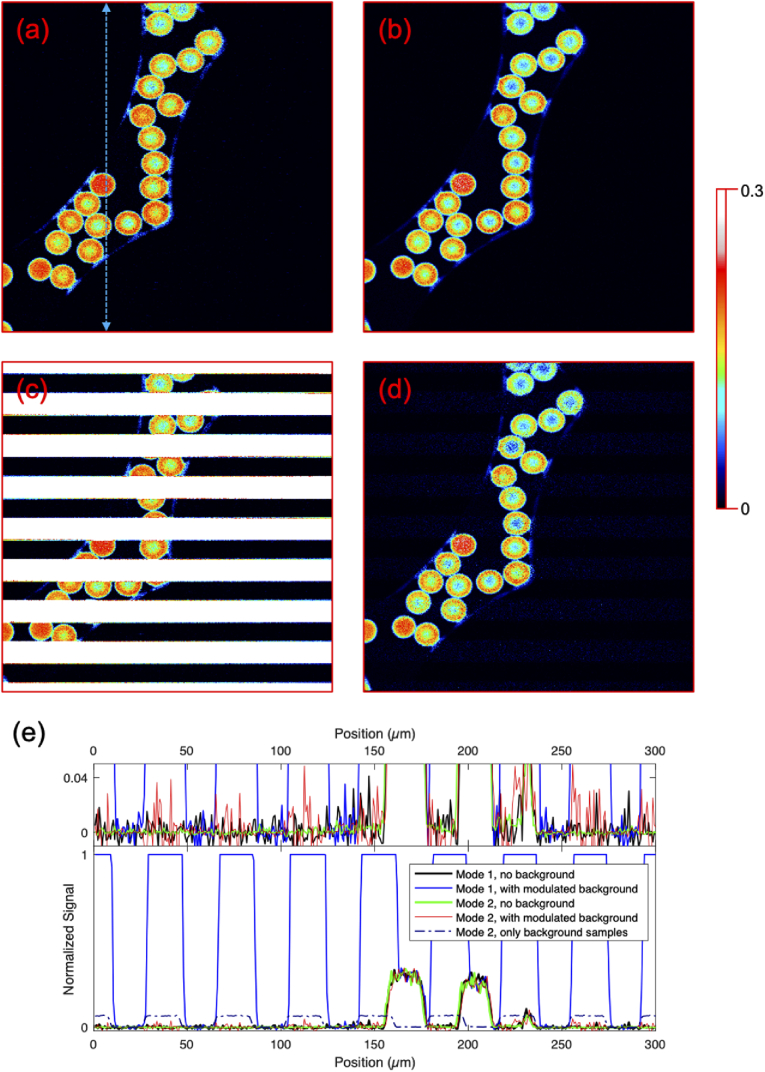
Two-photon excitation induced fluorescence from Dragon Green beads. (a) and (b) Image obtained when imaging with the first and second acquisition mode, respectively, under normal imaging conditions. (c) and (d) Images obtained when imaging with the first and second acquisition mode, respectively, with a 20 Hz modulated laser beam illuminating the sample from below. While the image in panel (c) has bright saturated stripes caused by the light detected from the green pump laser, the image in panel (d) exhibits almost no artifacts caused by the green laser light. Each of the 400×400 pixel images was recorded in 400 ms, and shows an area of 300×300 µm^2^. The false color scheme was chosen to highlight small signal details. (e) Cross-section from the four images shown in panels (a)–(d), at the location denoted by the dashed blue double-arrowed line in panel (a). (black– (a), first mode, and, green – (b), our method, both no background illumination; blue – (c), first mode, and, red – (d), our method, both with background illumination). The dark blue dash-dotted line shows the corresponding background measured from the background samples in the data-stream used to generate panel (d). Note that the dark noise in the black and blue traces is comparable to the noise in the regions in the red trace where background was subtracted (upper panel).

We have imaged a sample of fluorescent beads with and without additional illumination by the 20 Hz modulated green pump laser (acting as a source of artificial background) from below the sample using the two above-described data-acquisition modes. Without the green laser illumination both data-acquisition modes yield basically the same image (Fig. 4(a) was taken with the first and Fig. 4(b) with the second acquisition mode). Slight differences between the two images taken with the different acquisition modes are consistent with differences in consecutive images taken with the same acquisition mode. The observed noise on the fluorescence from the beads is the same in both images.

With the 20 Hz modulated green laser illuminating the sample from below, the image acquired using the first data-acquisition mode exhibits bright stripes where the image is saturated (the signal is clipped at the ADC range maximum), the appearance of those stripes exactly coincides with the time-periods when the green pump laser illuminates the sample from below, as it is expected, shown in Fig. 4(c). The second data acquisition mode allows subtracting the signal due to the modulated green light from each measured signal value, and the image acquired with this mode does not exhibit the bright stripes, see Fig. 4(d). When only the signal samples are used to form the image, the stripes (background) are reduced in intensity (up to a factor 14), compared to the first data acquisition mode in which the signal from the PMT is integrated over a timespan covering several laser pulses. This reduction in background intensity may be sufficient to allow removing the stripes using post-processing algorithms, but post-processing will be more challenging when time-dependent changes in fluorescence (for example of calcium sensitive dyes or genetically encoded calcium indicators) are measured, and in situations where relatively few pixels are expected to show no signal.

Figure 4(e) shows a cross-section of the same vertical line – indicated by the blue dashed double arrow in Fig. 4(a) – from the four images in Figs. 4(a)–4(d) together with the background integrated along the horizontal direction obtained with the second acquisition mode. Additionally, supplemental Fig. S1 shows a cross-section at a different position to highlight the first and second acquisition modes yield the same images and relative intensities. From Fig. 4(e) it can be seen that with our method, the background is successfully subtracted. A close examination of the signal levels in the areas where no fluorescent beads are located shows that the dark signal measured with the PCI-6110 has about the same fluctuations (noise) as the areas of the image taken with our method where the background light was present. The dark noise in the image taken with the second data-acquisition mode (without background light) is a factor of 5 lower than in the images taken with the PCI-6110 card. In other words, our method significantly reduces the signal-to-background ratio both in the presence and absence of background light. In the first acquisition mode the zero-signal level needs to be determined through evaluation of the images, and is sensitive to electronic drifts, e.g., due to temperature changes of the electronics. Our method automatically obtains the correct zero-value for the images, independent of possible offsets in the measured signals, because the background is subtracted in real-time.

The reduction in background level can be determined through comparison of the two images in Figs. 4(c) and 4(d) (Supplemental Fig. S2 shows the same data with the color scale spanning the dynamic range of acquisition mode 1), and Fig. 4(e). Figure 4(e) shows that the presence of the green laser illumination causes a higher noise in Fig. 4(d). With this noise, we can also estimate a global uncertainty in the background level. Because the green laser illumination in Fig. 4(c) saturates the image, the background level in the first acquisition mode is underestimated. Using these two observations, a lower limit for the background reduction can be determined. From a comparison of Figs. 4(c) and 4(d) we estimate the lower value for the background suppression ratio of our method is 3000-fold for the green laser illumination power used. Supplemental Fig. S3 shows images taken with the power of the green laser adjusted to not clip the ADC, in this case the image taken with acquisition mode 2 yields similar background suppression to the case where the green illumination saturated the detection.

## 4. Discussion

We have presented a simple and cost-effective scheme for real-time background (and stray-light) suppression to facilitate implementations of simultaneous all-optical photo-stimulation and two-photon fluorescent imaging. Our method relies on having a signal sampling rate (and instrument response bandwidth) much higher than the laser pulse repetition rate, and the use of fluorescence markers with a fluorescence decay time much shorter than the time interval in-between two laser pulses. For this realization we have used an ultrashort all-fiber laser operating at 4 MHz repetition rate. Providing the peak intensity of the two-photon excitation pulse is below saturation (of the fluorophore excitation) and/or photodamage threshold, another advantage of the low-MHz repetition rate (while using the same average power as used at higher repetition rates) is that it allows for the implementation of a detection scheme using only a single laser pulse per pixel, maximizing the signal level and scanning speed [38]. The AOM in the laser can be operated up to ∼40 MHz, thus can allow to tune the laser repetition rate up to 29 MHz (transmitting every second pulse from the oscillator). It can also operate in a ‘continuous’ mode allowing the laser to operate at the 58 MHz repetition rate of the oscillator. The 4 MHz repetition rate of the laser was chosen because the delay generator card used to generate the AOM driving signal limited the trigger rate of the AOM to maximally 5 MHz. Alternative delay generator cards are available commercially that make it possible to achieve tunability up to much higher repetition rates. High laser repetition rates (>50 MHz) have the drawback that, since most fluorophores have a few-nanosecond excited state lifetime, the fluorescent emission will not have completely ceased when the next pulse arrives at the focus and the background cannot be accurately measured.

Because of the need to acquire multiple measurements in between two subsequent laser pulses, the 4 MHz laser repetition rate allows to use a moderately high sampling frequency (tens of MS/s) which allows integrating most fluorescence signal after the pulse excitation into one digital sample. Implementation of our approach is straightforward with moderately higher repetition rates, e.g., up to 20 MHz, using similar data acquisition hardware, i.e., with a maximum sample rate of 125 MS/s. Higher repetition rates (> 20 MHz) will require a higher sampling rate, and much faster electronics to ensure optimal signal processing. It is worthwhile to note that few MHz repetition rate (between 1 and 10 MHz), high energy pulses are considered optimal for deep tissue imaging [40,41]. It may also be noted here that imaging using an 8 kHz resonant mirror at 512×512 pixel resolution (yielding ∼30 Hz frame rate) results in a pixel rate of about 8 MHz.

Besides for multi-photon fluorescence, the method presented here is also compatible with other techniques based on nonlinear optical effects such as coherent anti-Stokes Raman scattering (CARS), second harmonic generation (SHG), or third harmonic generation (THG), which inherently satisfy the requirement of showing a response time much faster than the time in between two subsequent laser pulses. Thus, a time-gated signal acquisition can be achieved, as well as an independent measurement of the background light level. Sampling rates much higher than the laser repetition rate are possible with affordable commercially off-the-shelf electronics even for commonly used pulse repetition rates for fluorescence excitation in nonlinear imaging (typically 80 MHz or below).

Our method functions in a way equivalent to lock-in amplification, but its realization does not require any additional hardware provided the fluorophore lifetime is compatible with the repetition rate setting of the system and the FPGA is properly programmed. Compared to our method, lock-in amplification limits the imaging speed that can be achieved, because the frequency filtering of a lock-in amplifier requires signal acquisition over several periods (laser pulses), i.e. lock-in amplification is not compatible with image-acquisition using only a single laser pulse per pixel. Our method explicitly allows for this, as the background is measured shortly (few tens of ns) after or before the laser pulse arrives and the fluorescence signal can be expected. This restricts the tolerable background signal to only contain frequencies considerably lower than the laser repetition rate, however, it is highly unlikely that background or stray-light with higher modulation frequencies will be occurring. The real-time background suppression approach we present here can also be implemented in confocal microscopes where the cw excitation light is appropriately modulated, for example with an AOM.

When combining multiphoton imaging with multiphoton optogenetic control, we identify three different experimental scenarios, each with different implications for the implementation of our approach:

First, the laser for optogenetic stimulation has a pulse repetition rate comparable to, or higher than the ADC sampling rate (and thus much higher than the imaging laser repetition rate). In this case, artifacts caused by the optogenetic stimulation appear similar to artifacts caused by a continuous wave laser, and no modification to the implementation of our method presented here is required.

Second, the laser for optogenetic stimulation has a pulse repetition rate comparable to, or lower than the laser used for imaging, and is synchronized to the imaging laser. A similar situation is used in experiments combining two- and three-photon deep-tissue imaging [42]. Since the relative timing of the two lasers can be controlled by the experimenter, artifacts due to the optogenetic stimulation can be avoided. For the timing and choice of the background samples both the imaging laser and the optogenetic stimulation pulse timing need to be taken into account.

Third, the laser for optogenetic stimulation has a pulse repetition rate significantly lower than the ADC sampling rate and is not synchronized to the imaging laser. Because the repetition rate is lower than the ADC sampling rate, the artifacts cannot be treated similar to the first case. Because the relative timing with respect to the imaging laser varies, artifacts cannot be treated similar to the second case either. In order to minimize artifacts, it is helpful to record the timing of the optogenetic stimulation pulses, it may not be possible to remove artifacts for all pixels, but it will allow to reduce the number of pixels affected compared to for example the situation in the experiments by Yang et al. [13]. In this last scenario specifically, but also in general, it is worthwhile to save not only the corrected signal samples, but also the raw signal and background samples, in order to improve post-processing and to increase confidence in the extracted transients.

In super-resolution microscopy, more precisely in gated STED microscopy [34], time-gated fluorescence detection is also used. However, the intention is not to detect and separate fluorescence and stray-light that are not correlated with the subject to be imaged (in the context of gated STED, uncorrelated background refers to background correlated exclusively to the STED beam), but to suppress the detection of early fluorescence (which occurs while the depletion beam has not yet completed its function) and upconverted light induced by the STED beam, to enhance the imaging resolution. This requires a different sampling strategy than what is applied in our method.

Two-photon excited fluorescence imaging in the presence of a fluctuating strong background signal (which spectral peak coincides with the emission spectrum of Dragon Green fluorescent beads) in this proof-of-principle experiment was performed using two different data acquisition modalities and sampling rates. Using a conventional 1 MS/s card in combination with our 4 MHz Yb:fiber laser system, our method for background subtraction could not be implemented because the requirement of oversampling the signal was not satisfied. The recorded image [Fig. 4(c)] presented periodical white stripe regions due to the PMT signal being higher than the ADC input range maximum when the modulated 532 nm beam was illuminating the sample from below. When a fast ADC (58.2 MS/s, max 125 MS/s) combined with an FPGA was used for data collection, under the same 532 nm illumination conditions, background suppression was possible and a reduction in background of several orders of magnitude compared to the conventional acquisition mode was achieved. Sampling rates on the order of 80 MS/s are common in state-of-the-art commercial, as well as custom-built, two-photon microscopes equipped with commercial ultrafast lasers (e.g. 80 MHz Ti:sapphire oscillators). Since no oversampling is provided, this situation is comparable to our first case. Even if oversampling can be achieved, a more fundamental limitation that is not directly associated with the ratio of sampling rate and pulse repetition rate is the limitation in the amount of average power that the sample could handle before photodamage occurs. Since the average power is limited, higher repetition rates imply lower peak intensities on the sample, which will turn into lower signal per pulse and the requirement to increase the PMT gain. The increased PMT gain will also translate into higher sensitivity of the PMT to background/stray-light signals. Operating at lower repetition rates as compared to traditional high repetition rate sources, and having the possibility of tuning the repetition rate of the excitation source in combination with the implementation of the method for fluorescent signal imaging acquisition presented here has several advantages when it comes to optimizing imaging acquisition speed, improved background suppression, as well as for improving signal-to-noise ratio. Moreover, as compared to implementations of background subtraction using lock-in amplifiers our method does not require a reduction of the imaging speed, and data acquisition with one pulse per single pixel is possible.

In data acquisition mode 2, out of the 14 samples per laser pulse, we selected 1 sample with fluorescence signal, and 2 with background. We selected only two background samples, since due to the particular design of the TI ADC we observed electronic ringing and small electronic signal reflections in several of the other samples. The two samples we selected were the samples that were not compromised by these effects. No such problems were reported when a direct current-coupled ADC (such as for example the NI 5732 or NI 5734 digitizer) was used [42], and with such a digitizer more than 2 background samples can be used. The use of 2 background samples reduces the noise (where background light is present) by a factor of ∼1.2 over the use of only one background sample.

In some cases, it may be possible to employ our method while using an 80 MHz pulse train for excitation of the fluorescent reporter, for example when a very bright fluorophore with a very short fluorescence lifetime (<1 ns), is used. Even in this case, reducing the repetition rate, for example using an AOM, can be beneficial, for example to reduce the average power delivered on the sample or improve the signal-to-noise ratio. When reducing the repetition rate by a factor *N*, while maintaining amount of (time-integrated) fluorescence constant, the same number of fluorescence photons is generated by one pulse instead of by *N* pulses. As a consequence, the PMT bias voltage can be reduced, thereby improving the tolerance to background light.

In conclusion, high fidelity signal recording was achieved, due to very high discrimination between the two-photon fluorescent signal generated by the imaging light and signal originating from an uncorrelated source, resulting in images virtually free of background artifacts. Our method strongly reduces background artifacts in the fluorescence signal recording, by several orders of magnitude, and offers a path for simultaneous optical activation and multiphoton microscopy read-out of neural activity. This presents an improved solution compared to restricting application of the photo-stimulation beam during the fly-back time of the scanning beam [5,6], or post-processing analysis removing image frames affected by photo-stimulation artifacts [13,27,30]. Within some experimental constraints (regarding the fluorescence lifetime of the fluorescent reporter, and photo-activation by the imaging laser), our approach offers a path for simultaneous photo-stimulation and multiphoton (two-photon) fluorescent imaging with more flexibility when choosing the type of fluorescent reporter and optical stimuli, and enables experiments under ambient light conditions where needed.

## Notes

See Supplement 1 for supporting content.

## References

[1] Stosiek C. Garaschuk O. Holthoff K. Konnerth A., “In vivo two-photon calcium imaging of neuronal networks,” Proc. Natl. Acad. Sci. U. S. A. 100(12), 7319–7324 (2003). 10.1073/pnas.1232232100 12777621 PMC165873

[2] Helmchen F. Denk W., “Deep tissue two-photon microscopy,” Nat. Methods 2(12), 932–940 (2005). 10.1038/nmeth818 16299478

[3] Göbel W. Helmchen F., “In vivo calcium imaging of neural network function,” Physiology 22(6), 358–365 (2007). 10.1152/physiol.00032.2007 18073408

[4] Yang W. Yuste R., “In vivo imaging of neural activity,” Nat. Methods 14(4), 349–359 (2017). 10.1038/nmeth.4230 28362436 PMC5903578

[5] Reiff D. Plett J. Mank M. Griesbeck O. Borst A., “Visualizing retinotopic half-wave rectified input to the motion detection circuitry of Drosophila,” Nat. Neurosci. 13(8), 973–978 (2010). 10.1038/nn.2595 20622873

[6] Schnaitmann C. Haikala V. Abraham E. Oberhauser V. Thestrup T. Griesbeck O. Reiff D., “Color processing in the early visual system of drosophila,” Cell 172(1-2), 318–330.e18 (2018). 10.1016/j.cell.2017.12.018 29328919

[7] Häusser M., “Optogenetics: the age of light,” Nat. Methods 11(10), 1012–1014 (2014). 10.1038/nmeth.3111 25264778

[8] Emiliani V. Cohen A. Deisseroth K. Häusser M., “All-optical interrogation of neural circuits,” J. Neurosci. 35(41), 13917–13926 (2015). 10.1523/JNEUROSCI.2916-15.2015 26468193 PMC4604230

[9] Ju N. Jiang R. Macknik S. L. Martinez-Conde S. Tang S., “Long-term all-optical interrogation of cortical neurons in awake-behaving nonhuman primates,” PLoS Biol. 16(8), e2005839 (2018). 10.1371/journal.pbio.2005839 30089111 PMC6101413

[10] Packer A. Russell L. Dalgleish H. Häusser M., “Simultaneous all-optical manipulation and recording of neural circuit activity with cellular resolution in vivo,” Nat. Methods 12(2), 140–146 (2015). 10.1038/nmeth.3217 25532138 PMC4933203

[11] Jarvis S. Schultz S., “Prospects for optogenetic augmentation of brain function,” Front. Syst. Neurosci. 9, 157 (2015). 10.3389/fnsys.2015.00157 26635547 PMC4655245

[12] Ferenczi E. Tan X. Huang C., “Principles of optogenetic methods and their application to cardiac experimental systems,” Front. Physiol. 10, 1096 (2019). 10.3389/fphys.2019.01096 31572204 PMC6749684

[13] Yang W. Carrillo-Reid L. Bando Y. Peterka D. Yuste R., “Simultaneous two-photon imaging and two-photon optogenetics of cortical circuits in three dimensions,” eLife 7, e32671 (2018). 10.7554/eLife.32671 29412138 PMC5832414

[14] Ronzitti E. Conti R. Zampini V. Tanese D. Foust A. Klapoetke N. Boyden E. Papagiakoumou E. Emiliani V., “Submillisecond optogenetic control of neuronal firing with two-photon holographic photoactivation of chronos,” J. Neurosci. 37(44), 10679–10689 (2017). 10.1523/JNEUROSCI.1246-17.2017 28972125 PMC5666587

[15] Chen I. Papagiakoumou E. Emiliani V., “Towards circuit optogenetics,” Curr. Opin. Neurobiol. 50, 179–189 (2018). 10.1016/j.conb.2018.03.008 29635216 PMC6027648

[16] Soor N. Quicke P. Howe C. Pang K. Neil M. Schultz S. Foust A., “All-optical crosstalk-free manipulation and readout of Chronos-expressing neurons,” J. Phys. D: Appl. Phys. 52(10), 104002 (2019). 10.1088/1361-6463/aaf944 31057183 PMC6466639

[17] Boyden E. Zhang F. Bamberg E. Nagel G. Deisseroth K., “Millisecond-timescale, genetically targeted optical control of neural activity,” Nat. Neurosci. 8(9), 1263–1268 (2005). 10.1038/nn1525 16116447

[18] Deisseroth K., “Optogenetics: 10 years of microbial opsins in neuroscience,” Nat. Neurosci. 18(9), 1213–1225 (2015). 10.1038/nn.4091 26308982 PMC4790845

[19] Forli A. Vecchia D. Binini N. Succol F. Bovetti S. Moretti C. Nespoli F. Mahn M. Baker C. A. Bolton M. Yizhar O. Fellin T., “Two-photon bidirectional control and imaging of neuronal excitability with high spatial resolution in vivo,” Cell 22(11), 3087–3098 (2018). 10.1016/j.celrep.2018.02.063 PMC5863087 29539433

[20] Zhang F. Prigge M. Beyrière F. Tsunoda S. Mattis J. Yizhar O. Hegemann P. Deisseroth K., “Red-shifted optogenetic excitation: a tool for fast neural control derived from Volvox carteri,” Nat. Neurosci. 11(6), 631–633 (2008). 10.1038/nn.2120 18432196 PMC2692303

[21] Govorunova E. Sineshchekov O. Li H. Janz R. Spudich J., “Characterization of a highly efficient blue-shifted channelrhodopsin from the marine alga Platymonas subcordiformis,” J. Biol. Chem. 288(41), 29911–29922 (2013). 10.1074/jbc.M113.505495 23995841 PMC3795289

[22] McIsaac R. Bedbrook C. Arnold F., “Recent advances in engineering microbial rhodopsins for optogenetics,” Curr. Opin. Struct. Biol. 33, 8–15 (2015). 10.1016/j.sbi.2015.05.001 26038227 PMC4641784

[23] Gupta N. Bansal H. Roy S., “Theoretical optimization of high-frequency optogenetic spiking of red-shifted very fast-Chrimson-expressing neurons,” Neurophotonics 6(02), 1 (2019). 10.1117/1.NPh.6.2.025002 PMC6458485 31001567

[24] Wu J. Liu L. Matsuda T. Zhao Y. Rebane A. Drobizhev M. Chang Y. Araki S. Arai Y. March K. Hughes T. Sagou K. Miyata T. Nagai T. Li W. Campbell R., “Improved orange and red Ca^2+^ indicators and photophysical considerations for optogenetic applications,” ACS Chem. Neurosci. 4(6), 963–972 (2013). 10.1021/cn400012b 23452507 PMC3689190

[25] Dana H. Mohar B. Sun Y. Narayan S. Gordus A. Hasseman J. Tsegaye G. Holt G. Hu A. Walpita D. Patel R. Macklin J. Bargmann C. Ahrens M. Schreiter E. Jayaraman V. Looger L. Svoboda K. Kim D., “Sensitive red protein calcium indicators for imaging neural activity,” eLife 5, e12727 (2016). 10.7554/eLife.12727 27011354 PMC4846379

[26] Qian Y. Piatkevich K. McLarney B. Abdelfattah A. Mehta S. Murdock M. Gottschalk S. Molina R. Zhang W. Chen Y. Wu J. Drobizhev M. Hughes T. Zhan J. Schreiter E. Shoham S. Razansky D. Boyden E. Campbell R., “A genetically encoded near-infrared fluorescent calcium ion indicator,” Nat. Methods 16(2), 171–174 (2019). 10.1038/s41592-018-0294-6 30664778 PMC6393164

[27] Zhang Z. Russell L. Packer A. Gauld O. Häusser M., “Closed-loop all-optical interrogation of neural circuits in vivo,” Nat. Methods 15(12), 1037–1040 (2018). 10.1038/s41592-018-0183-z 30420686 PMC6513754

[28] Fajardo O. Zhu P. Friedrich R., “Control of a specific motor program by a small brain area in zebrafish,” Front. Neural Circuits 7, 67 (2013). 10.3389/fncir.2013.00067 23641200 PMC3640207

[29] Simpson J. Looger L., “Functional imaging and optogenetics in Drosophila,” Genetics 208(4), 1291–1309 (2018). 10.1534/genetics.117.300228 29618589 PMC5887132

[30] Accanto N. Chen I. Ronzitti E. Molinier C. Tourain C. Papagiakoumou E. Emiliani V., “Multiplexed temporally focused light shaping through a gradient index lens for precise in-depth optogenetic photostimulation,” Sci. Rep. 9(1), 7603 (2019). 10.1038/s41598-019-43933-w 31110187 PMC6527563

[31] Rickgauer J. Tank D., “Two-photon excitation of channelrhodopsin-2 at saturation,” Proc. Natl. Acad. Sci. U. S. A. 106(35), 15025–15030 (2009). 10.1073/pnas.0907084106 19706471 PMC2736443

[32] Connally R. Piper J., “Time gated luminescence microscopy,” Ann. N.Y. Acad. Sci. 1130(1), 106–116 (2008). 10.1196/annals.1430.032 18596339

[33] Yang W. Chen S., “Time-gated fluorescence imaging: advances in technology and biological applications,” J. Innov. Opt. Health Sci. 13(03), 2030006 (2020). 10.1142/S1793545820300062

[34] Vicidomini G. Moneron G. Han K. Westphal V. Ta H. Reuss M. Engelhardt J. Eggeling C. Hell S., “Sharper low-power STED nanoscopy by time gating,” Nat. Methods 8(7), 571–573 (2011). 10.1038/nmeth.1624 21642963

[35] Ronzitti E. Harke B. Diaspro A., “Frequency dependent detection in a STED microscope using modulated excitation light,” Opt. Express 21(1), 210–219 (2013). 10.1364/OE.21.000210 23388913

[36] Berezin M. Achilefu S., “Fluorescence lifetime measurements and biological imaging,” Chem. Rev. 110(5), 2641–2684 (2010). 10.1021/cr900343z 20356094 PMC2924670

[37] Fernández A. Jespersen K. Zhu L. Grüner-Nielsen L. Baltuška A. Galvanauskas A. Verhoef A., “High-fidelity, 160 fs, 5 µJ pulses from an integrated Yb-fiber laser system with a fiber stretcher matching a simple grating compressor,” Opt. Lett. 37(5), 927 (2012). 10.1364/OL.37.000927 22378441

[38] Prevedel R. Verhoef A. Pernia-Andrade A. Weisenburger S. Huang B. Nöbauer T. Fernández A. Delcour J. Golshani P. Baltuska A. Vaziri A., “Fast volumetric calcium imaging across multiple cortical layers using sculpted light,” Nat. Methods 13(12), 1021–1028 (2016). 10.1038/nmeth.4040 27798612 PMC5531274

[39] Akerboom J. Chen T. Wardill T. Tian L. Marvin J. Mutlu S. Carreras Calderón N. Esposti F. Borghuis B. Sun X. Gordus A. Orger M. Portugues R. Engert F. Macklin J. Filosa A. Aggarwal A. Kerr R. Takagi R. Kracun S. Shigetomi E. Khakh B. Baier H. Lagnado L. Wang S. Bargmann C. Kimmel B. Jayaraman V. Svoboda K. Kim D. Schreiter E. Looger L., “Optimization of a GCaMP calcium indicator for neural activity imaging,” J. Neurosci. 32(40), 13819–13840 (2012). 10.1523/JNEUROSCI.2601-12.2012 23035093 PMC3482105

[40] Charan K. Li B. Wang M. Lin C. Xu C., “Fiber-based tunable repetition rate source for deep tissue two-photon fluorescence microscopy,” Biomed. Opt. Express 9(5), 2304–2311 (2018). 10.1364/BOE.9.002304 29760989 PMC5946790

[41] Wang T. Wu C. Ouzounov D. Gu W. Xia F. Kim M. Yang X. Warden M. Xu C., “Quantitative analysis of 1300-nm three-photon calcium imaging in the mouse brain,” eLife 9, e53205 (2020). 10.7554/eLife.53205 31999253 PMC7028383

[42] Weisenburger S. Tejera F. Demas J. Chen B. Manley J. Sparks F. Martínez Traub F. Daigle T. Zeng H. Losonczy A. Vaziri A., “Volumetric Ca^2+^ imaging in the mouse brain using hybrid multiplexed sculpted light microscopy,” Cell 177(4), 1050–1066.e14 (2019). 10.1016/j.cell.2019.03.011 30982596 PMC6602798

