# Supplementary material for: Dynamic real-time subtraction of stray-light and background for multiphoton imaging
Source: Biomed Opt Express. 2020 Dec 14;12(1):288–302. doi: 10.1364/BOE.403255 (PMC7899518; doi:10.1364/BOE.403255)
Supplement: Supplementary file 1 [file boe-12-1-288-s001.pdf]

Supplemental Document

Biomedical Optics EXPRESS

Dynamic real-time subtraction of stray-light and background for multiphoton imaging: supplement

A. Fernández,[1,2,3,7] A. Straw,[4] M. Distel,[5] R. Leitgeb,[6] A. Baltuska,[2] and A. J. Verhoef[1,2,3,6,8]

[1] *IQSE and Department of Soil and Crop Sciences, Texas A&M University, 4242 TAMU, College Station, TX 77843, USA*
[2] *Photonics Institute, TU Wien, Gusshausstrasse 27-29/387, 1040 Vienna, Austria*
[3] *Centro Regional Universitario de Coclé, Universidad de Panamá, Penonomé, Coclé, Panama*
[4] *Institute of Biology I and Bernstein Center Freiburg, University of Freiburg, Hauptstrasse 1, 79104 Freiburg, Germany*
[5] *St. Anna Children's Cancer Research Institute, Zimmermannplatz 10, 1090 Vienna, Austria*
[6] *Center for Medical Physics and Biomedical Engineering, Medical University of Vienna, Währinger Gürtel 18-20/4L, 1090 Vienna, Austria*
[7] *alma.fernandez@tamu.edu*
[8] *aart.verhoef@tamu.edu*

This supplement published with The Optical Society on 14 December 2020 by The Authors under the terms of the Creative Commons Attribution 4.0 License in the format provided by the authors and unedited. Further distribution of this work must maintain attribution to the author(s) and the published article's title, journal citation, and DOI.

Supplement DOI: https://doi.org/10.6084/m9.figshare.13350671

Parent Article DOI: https://doi.org/10.1364/BOE.403255

Dynamic real-time subtraction of stray-light and background for multiphoton imaging: supplemental document

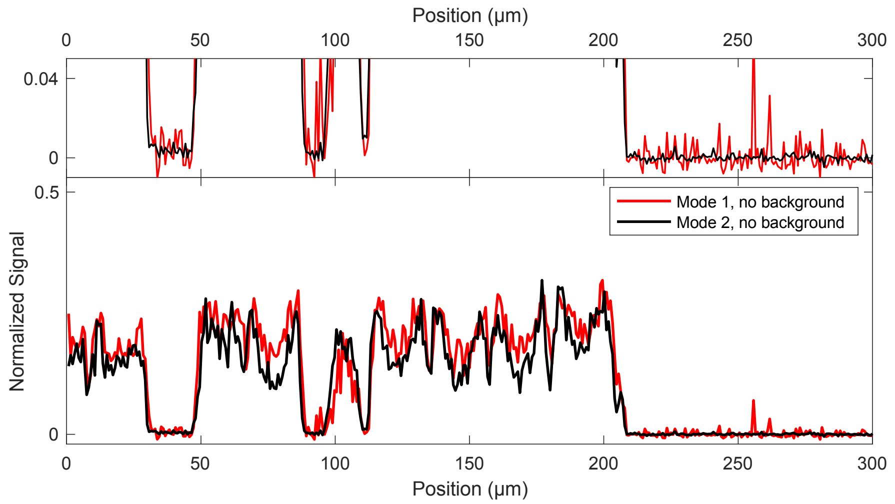

Fig. S1. Cross-section of Fig. 4(a) and (b) at a different position (vertically at the 184th pixel from the left) than shown in Fig. 4(e).

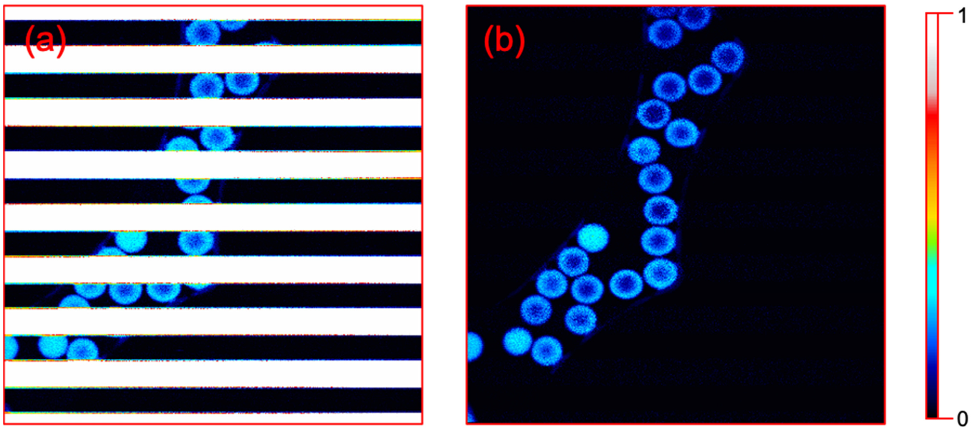

Fig. S2. Two-photon excitation induced fluorescence from Dragon Green beads. (a) and (b) Images obtained when imaging with the first and second acquisition mode, respectively, with a 20 Hz modulated laser beam illuminating the sample from below. The images are the displaying the same data as Fig. 4(c) and (d) in the main manuscript, but with the color scale adjusted to span the full dynamic range of the acquisition system in mode 1. Each of the 400×400 pixel images was recorded in 400 ms, and shows an area of 300×300 μm^2.

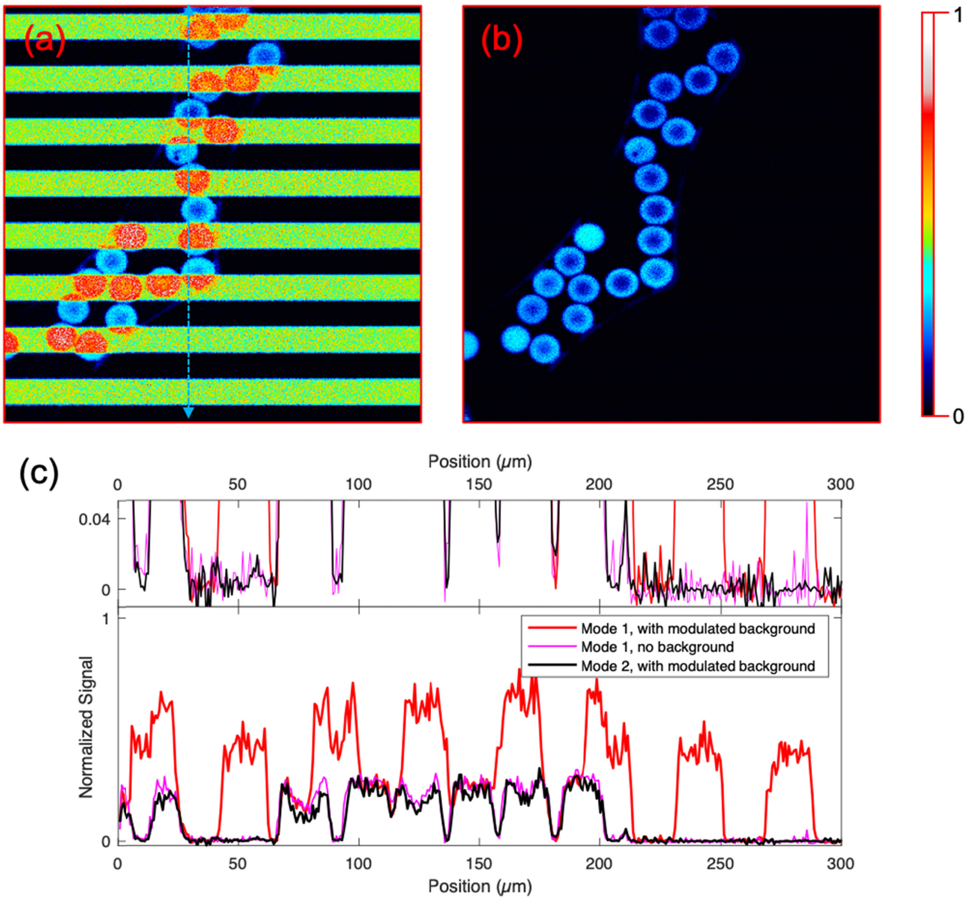

Fig. S3. Two-photon excitation induced fluorescence from Dragon Green beads. (a) and (b) Images obtained when imaging with the first and second acquisition mode, respectively, with a 20 Hz modulated laser beam illuminating the sample from below. The green laser power was reduced by ~3 times compared to the images in Fig. 4(c) and (d) in the main manuscript using a variable neutral density filter. While the image in panel (a) has bright stripes caused by the light detected from the green pump laser, the image in panel (b) exhibits almost no artifacts caused by the green laser light. Each of the 400×400 pixel images was recorded in 400 ms, and shows an area of 300×300 μm^2. The false color scheme was chosen to highlight small signal details and fully cover the full dynamic range of the detection system of acquisition mode 1. (c) Cross-section from the images shown in panels (a)–(b) and Fig. 4(a) in the main manuscript, at the location denoted by the dashed blue double-arrowed line in panel (a). (Magenta – first mode, no background illumination (Fig. 4(a), main manuscript); red – (a) first mode, and, black – (b), our method, both with background illumination). Note that the dark noise in the red and magenta traces is comparable to the noise in the regions in the black trace where background was subtracted (upper panel).
